# Modafinil in the media: Metaphors, medicalisation and the body

**DOI:** 10.1016/j.socscimed.2008.11.016

**Published:** 2009-02

**Authors:** Catherine M. Coveney, Brigitte Nerlich, Paul Martin

**Affiliations:** Institute for Science & Society, University of Nottingham, Nottingham, UK

**Keywords:** UK, Modafinil, Medicalisation, Human enhancement, Sleep, Metaphor, Media

## Abstract

This paper uses UK media coverage of the sleep drug modafinil to investigate the medicalisation of sleep at a conceptual level. Using metaphorical frame analysis we investigate the conceptual links created in media discourse between sleep and health, and the body and technology in the UK. Using this novel analytical tool we explore under what circumstances modafinil is constructed as a necessary medical treatment or a (il)legitimate performance enhancement and, how in this process, various images of the body are constructed. We found that media discourse on modafinil was structured through four types of sleep discourse: patient, sports, recreational, and occupational. Each discourse was built up around the specific deployment of three central metaphorical frames ‘war’, ‘commodity’ and ‘competition’ that acted to construct the biological body in a particular way. How the body was framed in each discourse impacted upon how modafinil use was portrayed in terms of therapy or enhancement and the level of engagement with a medical rhetoric. This had distinct normative implications strongly influencing the legitimacy afforded to modafinil use in each domain. We argue that medical authority acts to legitimise modafinil use for repair, restoration and relief of suffering, whilst being deployed to pass judgment on its use in bodies already perceived as functioning normally. This leads us to conclude that conceptually, the acceptability of ‘enhancement’ is strongly tied to context of use and intricately related to medical social control.

## Introduction

Sleep has long been amenable to pharmacological manipulation with various hypnotics available to induce quiescence. However, we now have the pharmacological means to ‘treat’ sleepiness with the development of wake-promoting drugs such as modafinil. Modafinil is a ‘eurogic’ drug that promotes arousal, or ‘calm wakefulness’ (Cephalon, 2007), enabling the user to be awake for days at a time. The effects of modafinil reportedly include a variety of other cognitive benefits, such as improving alertness, concentration, and memory (Turner et al., 2005). Since its emergence in 1998 as a treatment for narcolepsy, various states of somnolence have been redefined in (bio)medical terms and subjected to pharmacological and/or psychiatric treatment. Modafinil's license has been extended to cover excessive daytime sleepiness (EDS) associated with a wide range of conditions including chronic fatigue syndrome, cancer, and other sleep disorders such as restless legs syndrome (RLS), obstructive sleep apnoea syndrome (OSAS) and shift work sleep disorder (SWSD).

Because of its multitude of uses, modafinil can be used as a good case study to investigate the reception and uptake of these new technologies within popular culture, the role and function of medicine in attempts to control sleep (once considered a private corporeal form of existence) and the normative implications this might have.

Recent studies in the sociology of sleep have focused their attention on the medicalisation/healthicization of sleep (Williams, 2003; Williams & Boden, 2004) asking ‘Is sleep another chapter in the medicalisation story?’ (Williams, 2002: 173). In this context, the term ‘medicalisation’ is understood as a bi-directional and multi-faceted process (Conrad, 1992) through which human differences are transformed into pathologies, diagnosable disorders and treatable conditions. Alternatively, the concept of healthicization is applied when studying advances in lifestyle causes and behavioural interventions (Williams, 2002). Williams (2002) argues that sleep in general is increasingly associated with issues of health and well-being, while specifically the diagnosis and treatment of many sleep problems are falling under the jurisdiction of medicine. He argues that ‘a (prospective) medicalisation of sleep *disorders*…runs parallel with the more general healthization of sleep’ (2002: 195). The ‘medicalisation’ of sleep has been investigated sociologically at different levels and across a variety of sites: at the organisational level, with the creation of specialised sleep clinics (Moreira, 2006); the interactional level in, for example, the context of the doctor–patient relationship (Hislop & Arber, 2003); and at a conceptual level through media discourses and debates about sleep problems (Kroll-Smith, 2003; Seale, Boden, Williams, Lowe, & Steinberg, 2007; Williams, Seale, Boden, Lowe, & Steinberg, 2008a; Woloshin & Schwartz, 2006), and potential treatments (Williams, Seale, Boden, Lowe, & Steinberg, 2008b).

The media have been shown to provide a central forum for debates regarding issues relating to science, society, lifestyle, and most importantly, health and illness (Nerlich & Clarke, 2003). It is mainly through the media that the general public becomes aware of scientific advances, new therapies – especially in the UK where direct-to-consumer advertising is not permitted (Williams et al., 2008b) – and the social and ethical issues regarding their use and availability. Because the media operate at this interface between science and society, reporting on scientific advances and technological developments in specific ways, they are likely to play an important role in shaping public perceptions of new technologies and their value and applications.

Williams et al. (2008b) attempt to go ‘beyond medicalisation’ in their recent study of the social construction of modafinil in the British print media, drawing attention to ‘the limits of a solely or strictly medicalised interpretation of these issues’. Instead they interpret the way media debates and discourses are organised around non-medical uses and abuses of the drug as ‘articulation or amplification of a series of cultural anxieties about the *pharmaceuticalisation* rather than the medicalisation of alertness, sleepiness and everyday/night life’ (2008b: 13). They define ‘pharmaceuticalisation’ as the ‘transformation of human conditions’ into ‘pharmaceutical matters of treatment or enhancement’ which ‘overlaps with but extend far beyond the realms of the medical or the medicalised’ and ‘serves to further blur the boundaries between treatment and enhancement’.

The use of medical technologies and procedures for self-improvement raises more general concerns about where the limits to medical authority over the body lie and the blurring of boundaries between therapy and enhancement (Parens, 1998). Due to the complex relationship between medicine and enhancement, these two concepts cannot be demarcated with ease. In one sense, all therapies can be conceptualised as enhancements as they extend, increase or improve mind, body or performance (President's Council on Bioethics, 2002). Recently, biomedical enhancement has been conceptualised as operating in three distinct ways: in terms of normalisation, bringing the body in line with a cultural norm; the repair and restoration of lost functions; or the (il)legitimate improvement of performance (Conrad, 2007). Some bioethicists argue that not all enhancements can be considered therapeutic as they may take an individual ‘beyond health’ (President's Council on Bioethics, 2002). However, the distinction between therapy [restoring health or relieving suffering] and enhancement [the improvement or extension of capacities] is sociologically arbitrary (Conrad, 2007), as concepts of disease often lack clear boundaries and definitions of health are also socio-culturally constructed and flexible (Wolpe, 2002).

Sleep is a corporeal state, a lived and embodied experience (Meadows, 2005). An analysis of modafinil, a technology that can be used to correct, alter or interfere with the functioning of the body, must also consider cultural representations and conceptualisations of the body it is being taken into. In their analysis of newspaper coverage of modafinil in the military, Williams et al. (2008b) briefly discuss concerns raised in media discourse over how the body may be reconfigured through modafinil use. We pay more attention to this point, giving the framing of the body a greater role in our analysis, as we would argue that understanding the kind of bodies technology is working on or taken into plays an important role in elucidating how the technology in question is itself understood (Thacker, 2002). In this context it is important to understand what type of ‘bodies’ are implied by the various discourses around modafinil.

Acknowledging the multi-faceted nature of sleep and how sleep has been linked to health, well-being, performance, dangers and deficits in a multitude of ways, we focus on one aspect of the sleep story, namely the medicalisation of sleep and more precisely, the medicalisation of sleep at a conceptual level. The definition of medical norms through the existence of new scientific knowledge and/or new medical treatments may change perceptions of how the human body functions and, importantly, influence social and cultural expectations of how the body *should* function.

Previous work has examined the social construction of modafinil in the British print media using a thematic and interpretative analysis to reveal how modafinil is constructed in terms of its various uses and abuses (Williams et al., 2008b). In applying metaphor analysis combined with frame analysis to this area, we go beyond previous research to empirically investigate the discursive construction of these ‘uses and abuses’ in the media. We focus our analysis on the metaphorical frames used in media discourses and the conceptual links they create between sleep and health, and the body and technology. Using this novel analytical tool, we can explore under what circumstances modafinil is constructed as a necessary medical treatment or a (il)legitimate performance enhancement and how, in this process, various images of the body are constructed. This will allow us to assess to what extent we are seeing the medicalisation of sleep in different domains, what normative assumptions are embedded in discourse on modafinil, and to comment on the relationship between medicine, enhancement and cultural understandings of the body.

Questions we seek to address include: How is modafinil discursively constructed in the British print media? How does this influence the configuration and reconfiguration of the body in popular consciousness? How and where is ‘medicalisation’ deployed? And to what effect? What does this tell us more generally about cultural attitudes towards human enhancement?

## Methods

In this study we focus on newspaper articles to explore discourses surrounding the new sleep drug, modafinil. This is achieved through an in-depth analysis of the language used to describe and the conceptual metaphors employed to articulate the multiple uses of modafinil. We are interested in the messages, behavioural directives and bodily narratives that are being made available in the media, rather than how this information is received and understood.

Recent analyses of the construction of modafinil in the media have contributed to such an understanding (Williams et al., 2008b). However, in order to gain more fine-grained insights into how the media portray the uses and abuses of modafinil and its status in science and society, it is necessary to apply a method that can give access to discursively and cognitively more deeply embedded and sometimes hidden conceptualisations. Metaphor analysis combined with frame analysis provides such a tool and has been used successfully in media studies and science and technology studies (STS) in recent years to reveal hidden agendas, ideologies and beliefs about emerging technologies, policy controversies and issues of health and illness (Wallis & Nerlich, 2005).

Media coverage of medicines and other health products is often framed by ‘stock stories’ (Seale, 2002) in part generated though metaphor. In such stories the metaphorical systems used to describe illness, disease and the body are all important linguistic choices which can reveal deep social anxieties about the control of health and the control of society. According to Lupton (1994: 78) ‘representations of the ill body are inherently political, seeking to categorise and control deviancy, valorise normality and promote medicine as wondrous and ever-progressive.’ The way metaphors are used in the media to draw parallels between seemingly unrelated concepts and to make the novel or unfamiliar appear familiar is therefore an important aspect of analysing media data. Metaphors not only structure different forms of discourse but organise ways of living (Lakoff & Johnson, 1980) and metaphor analysis therefore contributes an important dimension to research on the medicalisation of sleep.

**Fig. 1:**
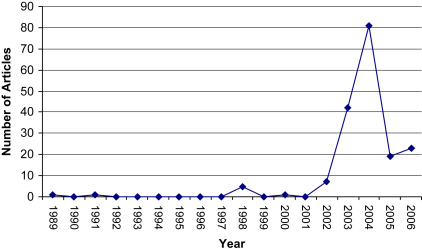
UK media coverage of modafinil/Provigil 1989–2006.

Using the search terms *modafinil* and *Provigil*, Lexis Nexis Professional and BBC news and Sport Online were used to locate news articles, from first loading (1989) to December 2006, appearing in UK broadsheets or on a UK news website (Fig. 1). Duplicates were eliminated as well as articles in which modafinil was not central to the story. The remaining corpus consisted of 53 broadsheet articles and 24 BBC news stories. We focused on broadsheets for pragmatic reasons; they have accessible archives and are predominantly textual resources. More visually orientated media would be more difficult to analyse using this approach. News articles published on the web, accessed through the BBC News and Sport online archive, were included in the study as recent research shows that the Internet is also an important site through which people access current news stories and information about health and well-being (Fox & Rainie, 2002).

We undertook an iterative analysis process, re-reading and coding the corpus of articles, generating themes, and cross-checking these through discussions between authors. Thematically related parts of the embedded analysis in each data source were grouped together. The authors discussed the coding of articles with each other, ensuring inter-researcher reliability of interpretation and enhancing analysis. The articles were first categorised according to their main theme(s). Four main themes emerged, related to four ‘discourses’ in which modafinil use was discussed; patient discourse (focus on treating a sleep disorder); sports discourse (focus on the use of modafinil by athletes); occupational discourse (focus on military, shift workers, students); recreational discourse (focus on leisure or general use).

During the next stage of analysis articles were read and re-read to isolate sub-themes and central metaphorical concepts in order to reveal emerging frames and their distinctive features (Entman, 1993). ‘Frames’ organize thought and package complex information by focusing on certain interpretations over others. From a cognitive linguistic viewpoint, the correspondence between two frames can be established via a conceptual metaphor, that is, a mapping between a source (mostly physical) and target (mostly abstract) domain. Conceptual metaphors shape how we see and act in the world on an ontological level and how we know and understand the world on an epistemological level. An example of a conceptual metaphor is ARGUMENTS ARE WAR. Utterances based on such a mapping between two domains might be, for example, “She shot down my argument”, “He lost the argument” and so on. These expressions are not only based on an abstract mapping between the two domains, but exploit the wider ‘war frame’ which involves, for example, the use of weapons, winners and losers.

Sections of text containing conceptual metaphors were systematically isolated from each article and related expressions grouped together to enable a detailed, even quantitative, study of their linguistic features and implicit value judgements (refining methods proposed by Kövecses, 2006; Lakoff & Johnson, 1980; Schmitt, 2005). Epistemologically and ontologically metaphor research rooted in the Lakovian paradigm is opposed to ‘objectivism’. It recognizes the importance of subjective and cultural meanings in all human experience especially with relation to the human body.

The extent to which each metaphorical frame was found in each of the four sleep discourses was quantified to gain an overall picture of the way such metaphoric expressions framed the media discourses. We identified many different types of metaphorical expressions. However, we focus our analysis on the use of the three most prevalent metaphorical frames used to varying degrees across the four discourses; ‘WAR’ [fighting sleepiness], ‘COMMODITY’ [trading sleep] and ‘COMPETITION’ [beating sleepiness]. We then determined the evaluative orientation of each sleep discourse by counting how often a metaphorical expression was used in a positive or negative way. This enabled us to assess the extent to which modafinil use was portrayed as legitimate or illicit.

In the following section we describe the three major metaphorical frames in detail before moving on to discuss how they were deployed in each of the four sleep discourses.

## Metaphors and frames

In this section we describe three distinct metaphorical frames that were used to structure media discourse on modafinil and analyse how they enable the body, corporeal states and the use of drugs to be constructed in specific ways. We show how the metaphorical frames are built up around a central metaphorical concept that frames the use of modafinil within a culturally available narrative. Metaphorical frames are not based solely upon salient metaphors, but around particular and sometimes inconspicuous metaphoric expressions that enable discourse on pharmaceutical intervention in the sleep–wake cycle to be articulated in a specific way. Each article contained some, but rarely all, components of one or more metaphorical frames. Our approach, however, is based upon an analysis of how the metaphorical frames are built up and used to structure discourse across the media sample as a whole, rather than in individual articles.

### Frame 1. War

The war frame was based around the use of military metaphors that constructed the ‘body as a battleground’ in which modafinil was launched to ‘combat’ ‘attacks’ of sleep. An analysis of the components of the war frame revealed that four concepts of war were drawn upon by the media; that of an enemy or injustice; the strategic war plan and events of the battle; personification of victims and heroes; and purpose or desirable outcome. Sleep was described as a ‘killer’, a dangerous ‘enemy’ that could ‘attack’ or ‘strike’ at any time. People with sleep problems were portrayed as the ‘victims’ of this metaphorical war, living through a constant ‘battle’ struggling to ‘fight’ off ‘sleep attacks’. Modafinil was framed in heroic terms being constructed as something that could be ‘launched’ to both ‘combat’ sleep and also as a type of armour that could prevent further ‘attacks’. Through this framing the story ends with modafinil giving those with sleep problems control back over their body, in a sense to win the battle and achieve victory over their illness.

Military metaphors used in this way allowed EDS to be framed as dangerous, and in the majority of cases, modafinil was constructed as a safe and effective treatment for this condition. By enabling individuals to stay awake during the day and sleep at night, pharmaceutical treatment was represented as restoring normal sleep patterns and thus providing the means through which one could lead a normal life. War frames are popular in many discourses on health and disease. They provide a strong focus and a moral imperative to use the means available to ‘help’ the individuals in question. The war frame allowed for medical and social uses of modafinil to be demarcated through the concept of ‘abnormality in functioning’. In discourse structured through this frame, the diseased, injured or abnormal body was transformed, via the act of taking modafinil, into a ‘normal’ body. Modafinil use was constructed as a positive action to restore impaired bodily functions, whether they arose as result of biological lesions or social factors. In both cases, medicine was given authority over the sleep–wake cycle.

When modafinil was perceived to be entering a ‘normal’ body in which there was no battle to be fought (i.e. in individuals without sleep problems), its usage was framed as a type of ‘enhancement’ falling outside the remit of medicine. In such instances, war frames were used to argue against the use of pharmaceuticals to ‘fight’ sleepiness. Individuals taking modafinil outside of medical authority became the villains of the piece, abusing this medicine for ‘lifestyle’ purposes. Concerned ‘scientists’, the new heroes, were used to voice fears of the dangers posed by unmonitored or uncontrolled use of this medical technology that might find its way into the wrong hands and the ‘wrong’ bodies.

### Frame 2. Commodity

The commodity frame was built up around mechanical and economic metaphors to include several aspects of a ‘commodity’: that it has a physical presence; can be renewed, replenished, diminished or depleted; and has an extrinsic value, so may be bought or sold. Within this frame the body was constructed as a machine, a set of parts, workings and systems. Sleep was often framed as a ‘fuel source’ required for ‘powering’ ones metaphorical engines. Individuals were described as needing to ‘fill up’ their bodies with enough sleep in order for them to remain ‘productive’ and ‘efficient’ and ‘function’ normally. However, ‘filling up with sleep’ was often framed as time consuming or ‘a waste of time’ and therefore a ‘luxury’ that many people could not ‘afford’, leaving them ‘running on empty’. Modafinil enters the story, again in a heroic form, a way to ‘keep going’, ‘a pharmaceutical miracle’ that could ‘change modern life’ or, more modestly, help us sleep ‘more efficiently’ when time is at a premium. Taking modafinil was therefore constructed as an alternative to sleep, an alternative method of providing ‘power’ by allowing sleep to be ‘traded’ for more time, and enabling individuals to adjust to the demands of a living in a 24/7 culture.

Situating stories about modafinil within a commodity framework links the novel and unfamiliar to pre-existing narratives regularly found in the media which present ‘sleep’ or a ‘good nights sleep’ as a consumer good (Williams, 2005; Williams & Boden, 2004). A plethora of different products selling ‘sleep’ are currently available, ranging from beds and pillows to herbal remedies and pharmaceutical products. Alternatively, products and strategies for maximizing alertness and energy are also widely available. According to Williams (2005: 165), ‘in the 24/7 society capitalism cashes in as both a disruptor and a guarantor of sleep’.

**Fig. 2:**
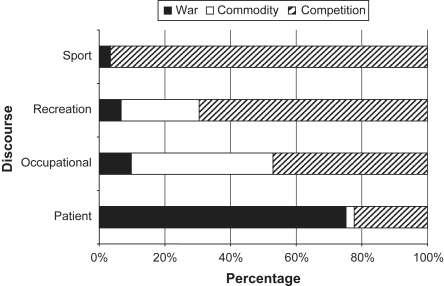
Prevalence of metaphorical frames in each discourse.

In discourse structured through a commodity framework, modafinil is constructed as a tool rather than a therapy, a way to technologically optimise the body/machine so it can function efficiently. The commodity frame was generally used to argue for pharmaceutical intervention in the sleep–wake cycle (75%, *n* = 57), constructing modafinil as an acceptable solution to the problem of excessive sleepiness in a 24/7 society. Commodity frames were mostly located within discourses of modafinil use in occupational and recreational contexts and often used in conjunction with competition frames (Fig. 2). The use of commodity frames provided an alternative way to articulate moral arguments for taking modafinil without necessarily having to demarcate the medical and social use of the drug. Through commodity frames wider societal concerns about the dangers of ‘normal’ sleepiness are brought into the discussion, allowing moral arguments for individual performance augmentation to be made on the grounds of both individual and public safety.

### Frame 3. Competition

The competition metaphorical frame was configured from several components of the competition source domain, including that of competitors; rules of the game; speed and distance; and that of a prize or goal. The competition frame was based around a metaphorical competition taking place between an individual and their body/bodily functions. Within this frame the body was viewed as malleable or ‘plastic’ and therefore open to biomedical augmentation, enhancement, improvement and design. Modafinil was constructed as a way to ‘beat’ sleep, an enhancement tool rather than a therapeutic that one could use to ‘eliminate the need for sleep’. Through the use of competition frames modafinil was often located within a ‘superhero’ storyline. In this well-known narrative, taking a drug (or other substance) transforms the individual in some way thus enabling performance beyond the norm. In this vein, the use of the technology was depicted as enabling an individual to ‘enhance’, ‘increase’, ‘improve’, ‘boost’ or ‘better’ their performance and capabilities outside of a ‘normal’ range, the literal outcomes of winning a metaphorical competition against the need to sleep.

Competition frames were used to argue both for (45%, *n* = 106) and against (55%, *n* = 123) pharmaceutical intervention in the sleep–wake cycle and were found across all four discourses (Fig. 2). The competition frame was often situated within articles discussing literal competitions where individuals would be depicted as not only competing internally against sleep, but also engaged in actual competitions on the sports field, workplace or in exams. This rhetorical strategy allowed parallels to be drawn between the two situations and similar moral judgements to be made. Using a drug to ‘beat sleep’ was often equated to cheating in the literal competition through the provision of an unnatural advantage that was condemned as illegal or unfair. Where a link to literal competition was more tenuous, metaphoric and other linguistic expressions were often used to compare modafinil to drugs such as caffeine, a substance already in widespread usage around the world to ‘beat sleepiness’. This rhetorical strategy sought to justify the use of modafinil in society through a context in which such a goal is conceptualised as a normal or everyday occurrence.

The competition frame enabled strong social values relating to competition and fairness to be articulated. The debate was focused at the level of the individual, with arguments based around freedom and autonomy and to what extent one should be allowed to choose what one does to one's own body. When expressed through this frame, the outcomes of taking modafinil were constructed as either individual improvement or individual detriment.

Overall, the three metaphorical frames were used to different extents across the four discourses in which modafinil use was discussed in the media (Fig. 2). Uncovering the underlying structure of media discourses through metaphoric frame analysis enables a deeper understanding of how different arguments are expressed and linked to specific sets of cultural values with distinct moral implications. War metaphors were related to ‘healing’, commodity metaphors to ‘efficiency’ and linked to discourses of ‘public safety’, whereas competition metaphors were related to ‘individual improvement’.

## Metaphorical framing of sleep discourses

In this section we move on to assess how the three central metaphorical frames were used to structure four types of discourses about the (il)legitimate use of modafinil in four domains of social life: the use by patients, for recreation, in the context of work and in sport. These discourses broadly relate to and overlap with the four key themes of ‘medical conditions’; ‘lifestyle choices’; ‘military operations’; and ‘sporting competition’ that have previously been identified as of importance (Williams et al., 2008b). Our analysis, by contrast, focuses on how the particular use of frames affects the boundary between medical and social constructions of pharmaceutical intervention in the sleep–wake cycle in these four contexts. We discuss the complex relationship between medicine and enhancement through consideration of the functions of the rhetoric of medical authority in the media discourse, the type of bodies being (re)constructed and the normative assumptions embedded therein.

### Patient discourses: abnormal bodies

**Fig. 3:**
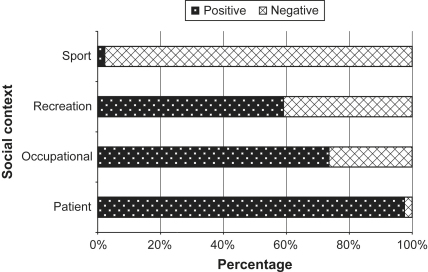
Positive/negative orientation of each discourse.

Patient discourses were predominantly structured through the war metaphorical frame (Fig. 2) and were overwhelmingly in favour of pharmaceutical intervention in the sleep–wake cycle (Fig. 3) as a method of maintaining or restoring a ‘normal’ body through the tools of medicine. The organisation of discourse around the concept of normality has the effect of not only describing how things are, but also inferring how they ought to be (Hacking, 1996). Patient bodies were designated as ‘abnormal’ and in need of correction or normalisation (see Fraser & Greco, 2005: 17) with pharmaceutical intervention constructed as a legitimate medical intervention in all instances. By giving the individual control back over their sleep–wake cycle, modafinil was framed as a chemical solution to restore the body to a normal level of functioning and allow the individual to be able to lead a more ‘normal’ life.

This resonates with a substantial body of social research into the use of metaphors in discourses relating to many different areas of medicine and disease (Riesfield & Wilson, 2004). Research in this area claims that metaphors can have a powerful influence on the practice of medicine and the experience of illness. The war metaphor is often prevalent in such discourses. According to Riesfield and Wilson (2004: 4025) ‘war has an exceptionally strong focusing quality and its images of power and aggression serve as strong counterpoints to the powerlessness and passivity often associated with serious illness’.

The metaphorical war frame was used to justify pharmaceutical intervention at both the individual and societal level, with the rare sleep disorder narcolepsy often the main point of reference through which moral reasoning about pharmaceutical intervention in the sleep–wake cycle was articulated. Interviews with narcoleptics frequently appeared in this discourse adding a human-interest dimension to the disorder and its treatment. Narcolepsy was described as ‘a disabling condition which interrupts studies, makes work impossible and destroys relationships’ (*The Independent*, 04/03/98). The treatment of narcolepsy with modafinil was constructed as a positive action, enabling the narcoleptic to overcome their disability and restoring the individual to a regular pattern of wakefulness during the day and sleep at night, as illustrated by the following example: “I am *fighting* a constant *battle* to stay awake…I know when I get tired, so I take a tablet at those times to prevent that tiredness”. (*The Daily Telegraph*, 01/10/02).

This type of framing was also observed at a societal level. Wake-promoting drugs were often represented as protecting society from the dangers posed by the problem of excessively sleepy individuals which might disrupt other people's ‘normal’ life. One headline in *The Independent* alerted readers to this problem by announcing that people with narcolepsy can ‘fall asleep at any time - even at the wheel of a car’ (28/09/00) and attacks of overwhelming sleepiness were blamed for ‘causing death on the roads’ (*The Times*, 05/03/98). According to advice offered by *The Times*'s resident medical doctor, EDS is a ‘dangerous condition and anyone with excessive daytime sleepiness should see their doctor’ (*The Times2*, 26/01/04). Here a direct normative stance emerges: people who have sleep problems *should* see their doctor and *ought* to take medication to regain normal functioning of their body so as to not endanger themselves or others. Therefore, in patient discourse medical authority was strongly linked to behavioural directives articulating a strong normative position: ‘normal’ bodies are desirable and can be produced through medicine.

### Sports discourses: natural vs. unnatural bodies

In the context of sport, competition metaphors (Fig. 2) were used to frame arguments against modafinil use (Fig. 3) and articulate concerns about fairness and legality. Modafinil was clearly seen as an enhancement technology and described as a ‘mood-enhancing psychostimulant’ that could ‘boost’ performance. Stories often equated modafinil with other drugs (e.g. steroids) that have been reportedly used as performance enhancing substances in sports. The use of modafinil by sportspersons was framed as deviant behaviour, whereby the power and tools of the medical profession were being used outside of medical authority by individuals to enable them to overcome their natural limitations and gain an ‘unfair advantage’ over others.

The sport discourse was characterised by strong moral judgements about modafinil use in this context. Taking modafinil in sport was represented as ‘cheating’, as devaluing the athletes' performance and as ruining their reputation. Competition frames constructed the act of taking modafinil in a sporting context as inducing an abnormal bodily state of prolonged wakefulness. Here the natural body was valorised with ‘naturalness’ equated to cultural conceptions of the normal, typical and regular (Fraser & Greco, 2005). It was argued that athletes should be ‘clean’, ‘natural’ and train hard as this is the only ‘fair’ and legitimate way to compete and to win. An example illustrating several elements of the competition frame and its normative implications can be found in the following quotation, in which an Olympic athlete condemns a fellow athlete's use of modafinil (this athlete later admitted taking modafinil and other banned substances as performance enhancers and testified before the Committee on Oversight and Government Reform):

‘People might wonder how she had the nerve to go in front of the world's media and offer an excuse like a sleeping disorder, but her nerve existed long before that. It went back to the first time she took drugs and lined up on the track, claiming to be clean and trying to win medals off people who have legitimately trained hard’. (*The Daily Telegraph*, 03/06/04)

The framing of modafinil through the competition frame as a way of overriding normal sleep was associated with strong negative normative values and acted to exclude medical narratives to describe sleepiness in this context. Therefore medicine was not given (or not claiming to have) any cultural authority over the sleep–wake cycle in this domain. However, the use of modafinil by professional athletes could also be considered as an occupational use of the drug. In addition, susceptibility to circadian rhythm disorders would almost certainly apply to this group whose working conditions involve travelling and competing across different time zones. Despite this, in the sport discourse, modafinil was portrayed as a ‘sleep disorder drug’ that had found illicit use in this context as an enhancement tool. This is interesting, given that the same drug is being taken to the same effect in each domain; the only difference being the context of use. Medicine was however still given rhetorical authority over the technology in question by the media, despite the fact it has found uses beyond the limits of medical control. Again, a relatively clear normative stance emerged: when there is no abnormality or impairment in functioning medical intervention *ought* not to take place as in these normal bodies this would not lead to healing the individual and, in addition, it would lead to ‘unfairness’ with regard to others in society.

### Occupational discourses: the body as a trading place

Through the combined use of commodity (40%, *n* = 42) and competition frames (50%, *n* = 52) in occupational discourses the body was represented as a trading place in which modafinil provided an alternative to sleep, and sleep could be traded for time. Individual bodies could be technologically optimised and adjusted to ‘stay alert’ or ‘stay awake longer’ and ‘function more efficiently’ in the modern workplace, making them more productive.

Within the occupational discourse we found a debate over the extent to which medicine has authority over the bodies of sleepy workers. Conflicting standpoints were evident as sleep problems resulting from working conditions were viewed as either a ‘normal’ part of working life and modafinil therefore a social intervention, or alternatively working conditions were seen as causing some degree of ‘abnormal functioning’, making it possible to justify modafinil as a medical treatment. Despite such inconsistencies, the way in which this discourse was framed through commodity and competition metaphors enabled justification for the drug to be sought through alerting readers to the dangers posed by a tired workforce (to both the individual and social body), rather than through a normative association with normal bodies.

Arguments in support of modafinil use in the workplace constituted three quarters of occupational discourse (Fig. 3). Many of these arguments were situated within ‘horror stories’ detailing the devastating consequences excessive sleepiness could have in the workplace. For example, excessive sleepiness was blamed for shocking incidents of ‘friendly fire’ in war zones and major disasters, such as ship wrecks and train crashes, were attributed to a tired workforce. As illustrated below, modafinil was positioned within these arguments as a type of ‘saviour’ that could be used not only to sustain the capability of the workforce but keep people alive and prevent accidents: “Called Modafinil, it has already been investigated by military organisations in France, the US, and Britain, where *keeping weary soldiers alert can prolong their lives*.” (*The Independent*, 10/07/97. Emphasis added). A second rhetorical strategy found in the occupational discourse to argue for the social use of modafinil was based upon normalising the idea of taking a performance enhancing substance at work: “American users describe in enthusiastic terms how the pill has enabled them to *stay awake* without the jitteriness and anxiety brought about by large amounts of caffeine” (*The Sunday Times*, 04/07/04).

Modafinil was compared to other drugs used in the workplace (e.g. caffeine) with claims made that modafinil ‘is already being used’ in this context as an ‘enhancement’ rather than as a ‘therapy’. Increasing sales of the drug were attributed to shift workers taking modafinil ‘off-license’ to ‘remain functional after a busy night’ (*The Times*, 02/07/05). In a military context, modafinil was more clearly demarcated as an enhancement technology with the ‘soldier–modafinil complex’ represented as a ‘cyborg fusion’ (Haraway, 1990), blurring the boundaries between body and technology (see Williams et al., 2008b). Soldiers on modafinil were constructed as being able to adapt to their environment and perform with maximum efficiency. Here, competition metaphors were used to frame the drug as a way of gaining a ‘military advantage’ (*The Independent*, 28/09/00), providing troops with an ‘extra edge’ (*BBC News*, 26/10/06) and allowing them to ‘feel more alert’ and function ‘better’ (*The Guardian*, 30/07/04) without needing to sleep. It was suggested that modafinil was a ‘better’ option than existing drugs said to be already used by the military, such as amphetamines, as it works ‘longer’, is ‘more effective’ and has ‘fewer’ side effects.

Around twenty-five percent of occupational discourse presented arguments against pharmaceutical intervention in the sleep–wake cycle (Fig. 3). These often drew upon potential detriments to health, including abuse and addiction, therefore demonstrating the moral judgements attached to taking drugs outside of medical authority in British culture. Opposition to modafinil use by the workforce was often justified at the level of individual safety and liberty, invoking fears of coercion and harms to individual health through the uncontrolled use of the ‘tools of medicine’. In one instance the commodity frame was used to describe soldiers' bodies as being ‘wired awake’ through modafinil, as if they were being coerced into prolonged wakefulness and forced to survive with little sleep. The voices of concerned doctors and scientists were used to criticise the non-medical use of modafinil blaming overwork or stress for excessive sleepiness at work. Using modafinil to prevent sleepiness was viewed as allowing people to ‘work harder and play harder’ drawing on fears of potential detriments to health with rest rather than pharmaceutical intervention put forward as a solution. Within the competition frame many of the arguments against the use of modafinil in a work-related context related to the ‘rules of the game’ component of the frame resulting in the normative arguments bearing great similarity to those evoked in the sport discourse. Furthermore, at a societal level questions were raised over the value of using drugs to improve performance. The costs of enhancement on a wider scale were evident here and included fears based on increasing competition in all areas of life and homogenising individuals into a norm influenced by current social and cultural standards.

To summarise, despite a high prevalence of medical rhetoric, justifications for the legitimate use of modafinil in this social context were generally sought through appeals to individual and public safety where the technology was framed in terms of its ability to protect society (the social body) from harm and danger. Normative questions emerged then around modafinil use on the boundary between ‘work’ and ‘lifestyle’ (with ‘normal’ work being on the borderline between the two).

### Recreational discourses: the ‘plastic’ body

Recreational discourses were structured through both competition and commodity frames (Fig. 2). Whereas societal issues dominated normative reflections in occupational discourses, the focus shifted to individuals and their lifestyle choices when modafinil use was discussed in a recreational context. Within this discourse the body was conceptualised as ‘plastic’ in the sense that it could be altered, changed, moulded, and designed. It was constructed as a site for optimisation and improvement, a commodity through which one could construct oneself. This understanding of the body fits into a paradigm of consumer culture that is based on an ideology of our ability to create and transform, in which one can choose both who one wants to be and how one wants to be.

In this context, arguments for and against pharmaceutical intervention in the sleep–wake cycle were given almost equal attention (Fig. 3). Within recreational discourses opposing viewpoints clashed over whether modafinil use should be viewed as a way of ‘trading sleep for more time’ and ‘improving ourselves’ by overcoming our evolutionary constraints or inducing an ‘unnatural’ and ‘abnormal’ state that could be detrimental to health and lead to widespread psychological addiction and drug abuse.

A natural/unnatural dichotomy was often used to frame arguments against the recreational use of modafinil and raise concerns over potential harms to health that could result from using pharmaceuticals to achieve an unnatural state of prolonged wakefulness. One article in *The Guardian* (25/04/06) used this dichotomy to criticise the whole idea of human enhancement, arguing that human enhancement is based upon the assumption that we are naturally inadequate. Other articles in the sample expressed fears that it may be difficult to ‘stay natural’ (*The Guardian*, 30/01/06) if drugs such as modafinil become readily available due to improved performance and increased competition, and ethical questions were raised about the use of drugs to gain advantages over others (*BBC News*, 13/07/05).

The majority (70%) of this discourse positioned the recreational use of modafinil as a social use of the drug. Pharmaceutical intervention in the sleep–wake cycle was represented as a way to reduce time spent sleeping, a method of potentially ‘eliminating sleep’ altogether and a tool to enhance ones cognitive abilities. Modafinil was tipped as the next ‘wonder drug’ to hit the UK with claims made that it could become the ‘pharmaceutical equivalent of the electric light bulb’ by ‘extending the waking day’ (*The Independent*, 04/03/98). However, and perhaps surprisingly, in around 30% of recreational discourses, the use of modafinil for ‘self-improvement’ was framed through the rhetoric of medicine. The competition frame allowed for the legitimate limits of medical authority to be debated within the media and the tensions between medical and social uses of technology to improve oneself to be expressed. An important aspect to this debate was the kind of bodies medical intervention was thought of as producing and whether this was a legitimate role for medicine to play in society.

For example, one article in *The Guardian* asks: “We improve ourselves via cosmetic surgery, why not also improve our brains?”(30/01/06). Such comparisons between modafinil [as a cognitive enhancer] with cosmetic surgery [a medicalised form of physical enhancement] were drawn to argue that medicine is already an institution through which we alter and enhance our normal bodies. Other arguments positioned such enhancement uses of modafinil outside of medical control referring to them as ‘lifestyle abuses’ of ‘sleep disorder drugs’.

The framing of modafinil use in this way resulted in the normative debate within recreational discourse being positioned at the individual level, with questions emerging about whether we *should* be allowed to alter ourselves using this technology. Fears and concerns surrounding potential consequences of individual augmentation were however often aimed at the social body.

With no impairment of functioning it appears more difficult to justify modafinil use without the moral imperative of restoring health. However, around one third of recreational discourses did construct modafinil use through the rhetoric of medicine. Interesting questions arise here regarding the role of medicine in self-improvement and the conceptual relationship between medicine and enhancement.

## Discussion and conclusions

In this article we explored representations of the wake-promoting drug modafinil in a corpus of UK media reports. Media reports on modafinil were categorised into four domains of discourse: patient, sports, occupational, and recreational, broadly relating to ‘key themes’ that previously have been shown to be of importance (Williams et al., 2008b). Each discourse was built up around the specific deployment of the metaphorical frames ‘war’, ‘commodity’ and ‘competition’ that acted to construct the body in a particular way. How the body was framed in each discourse impacted upon how modafinil use was portrayed in terms of therapy or enhancement and the level of engagement with a medical rhetoric. This had distinct normative implications strongly influencing the legitimacy afforded to modafinil use in each domain.

Both the patient and sports discourses were organised around the valorisation of ‘normal’ or ‘natural’ bodies in which relatively clear normative directives emerged: abnormal bodies and bodily functions (attributed to both biological and social factors) should be fixed through medical technology, whereas this technology should not be used in ‘normal’ bodies which do not need ‘healing’. This left room in the middle for debates regarding the legitimate role of medicine in society and the kind of bodies over which medicine is perceived to have authority. Occupational discourses were centrally concerned with notions of repair of lost functions or the prevention of harm – conceptually, an area medicine is increasingly moving towards with preventative medicine initiatives and health campaigns. Interestingly, in discussions of shift work, this was represented as not only a risk factor for other health problems, but one of the causal factors for a disorder in its own right, shift work sleep disorder (SWSD). At present only a small group of individuals with excessive daytime sleepiness (EDS) are thought to have SWSD. Are the media, then, promoting the medicalisation of work-related EDS through the expansion/extension of the disease boundaries for SWSD?

Although we found some evidence of such ‘disease mongering’ (Woloshin & Schwartz, 2006) by the media in the occupational discourse, the majority of articles bypassed the medical/non-medical debate altogether. The potential consequences of abnormal functioning (excessive sleepiness) were framed in such a way that the normative positions emerging in the discourse did not rely on the concept of normality nor the distinction between medical and social uses of the drug. Instead, justification was sought through appeals to wider non-medical narratives relating to both individual and public safety. However, medical rhetoric was prevalent in more critical aspects of this discourse, attending to potential negative consequences of using drugs outside of medical control. Despite the availability of a drug that can treat work-related sleepiness and the construction of a medical disorder (SWSD) to explain it, in its extreme form at least, a fully medicalised account of was not presented in this domain.

Cultural conceptions of ‘normality’ were also central in the recreational discourse where debates were situated around the use of modafinil for enhancement or improvement of ‘normal bodies’. We found that discourses concerning individual augmentation were often saturated with competition metaphors framing modafinil as an illicit ‘performance enhancement’. In these cases, the rhetoric of medicine was often used to argue *against* the application of modafinil in these situations, framing its usage as outside of medical control and therefore unauthorised. In other instances individual augmentation via modafinil was constructed as a medicalised form of self-improvement. Questions were raised regarding whether medicine should be used for enhancement purposes, and if this would be an abuse of medicine leading to the production of abnormal or unnatural bodies. Arguably, media constructions of modafinil as a medicalised ‘enhancement’, in the context of the commodification of medicine in a global healthcare market coupled with the rise of patient-consumers, could shape the demand for medical treatments to alter states of alertness, thus transforming medicine into a ‘vehicle for self-improvement’ (Conrad, 2007: 140). However, we found that in situations where no impairment or threat to society was identified in the media, there was a lack of moral imperatives to justify the enhancement of ‘normal’ bodies through medical intervention. Instead we found medical rhetoric was coupled with the moral obligation to restore health and normality, suggesting culturally at least, the Parsonian sick role prevails. This could however be due to the production of media texts where stories tend to be built up around the opinions of certified ‘expertise’.^1^

Media coverage of modafinil is complex, with medicalised discourses deployed in some contexts more than others. Using a new method and data set our study confirms to an extent Williams et al. (2008a) findings that at the conceptual level at least, ‘sleep is indeed another chapter in the medicalisation story’. However, discussions of modafinil for self-improvement revealed cultural anxieties about the future role of medicine in a culture of consumerism, and the kind of bodies medical technology *should* be used to alter.

When thinking about ‘uses and abuses’ of pharmaceuticals in terms of therapy and enhancement, it is actually very difficult to go ‘beyond medicalisation’ as Williams et al. (2008b) propose, as issues of ‘pharmaceuticalisation’ are undoubtedly bound up in processes of medicalisation and their normative connotations. We found a strong qualitative difference in the social and ethical issues raised in each domain of discourse. There are clearly different forms of enhancement, so how and where the technology was used became more important than its ‘biological composition’ (Conrad, 2007). At present it appears difficult to justify using medical technology for enhancement without the moral imperative of restoring health. In the case of new medical technologies such as modafinil that are approved for the treatment of specific conditions but can be used as enhancements for other capacities, medicalisation may in fact be a *requirement* in the legitimation of technological/pharmaceutical intervention whilst medical professionals act as ‘gatekeepers’ (Conrad, 2007) for their delivery. Medical norms play a role in setting social norms through the labelling of the abnormal. As such, further medicalisation of sleep at the conceptual level may lead to the expansion of medical social control through the creation of new expectations for bodies, behaviour and health.

As our analysis shows, through consideration of the normative issues allied to it, medical authority acts to legitimise enhancement for repair, restoration and relief of suffering, whilst being deployed to criticise enhancement in bodies already perceived as functioning normally. This therefore leads us to conclude that, conceptually, the acceptability of ‘enhancement’ is strongly tied to context and intricately related to medical social control.

The era in which we can pharmaceutically manipulate sleep and alertness it seems is upon us. Pharmaceutical companies are reportedly working on new technologies to alter sleep, thus creating further medicalised solutions to alter individuals to perform in line with cultural expectations and ideals, rather than prompt a change in the way we live our modern lives and the social conditions that have contributed towards the conceptualisation of sleepiness as a problem in the first place. However, if enhancement of normal bodies continues to be normatively constrained, a world in which one is free to technologically alter their need to sleep will remain a cultural biofantasy.

## Footnotes

1 Thank-you to one of the anonymous reviewers for bringing this point to our attention.
